# [2-(4-Chloro­phen­yl)-1,3-selenazol-4-yl]methanol

**DOI:** 10.1107/S1600536812048088

**Published:** 2012-11-28

**Authors:** Jichun Cui, Chuan Li, Yanling Qiao

**Affiliations:** a Shandong Provincial Key Laboratory of Chemical Energy Storage, and Novel Cell Technology, School of Chemistry and Chemical Engineering, Liaocheng University, Shandong 252059, People’s Republic of China

## Abstract

In the title compound, C_10_H_8_ClNOSe, the dihedral angle between benzene and selenazole rings is 11.4 (3)° and the hy­droxy­methyl group is bent from the selenazole ring, making a dihedral angle of 63.8 (3)°. In the crystal, mol­ecules are linked into inversion dimers by pairs of O—H⋯N hydrogen bonds. Roof-tile-like stacking of the mol­ecules along [010] [*b* = 4.5707 (4) Å] is observed, with the benzene and selenazole rings separated by a face-to-face distance of 3.57 Å and a mutual slippage of 2.85 Å.

## Related literature

For the synthesis of 1,3-selenazoles and their biological activity, see: Shafiee *et al.* (1979 ▶); Koketsu & Ishihara (2003 ▶); Geisler *et al.* (2004 ▶). For crystal structures of 1,3-selenazole derivatives, see: Shen *et al.* (2011 ▶); Shi & Zhao, (2007 ▶).
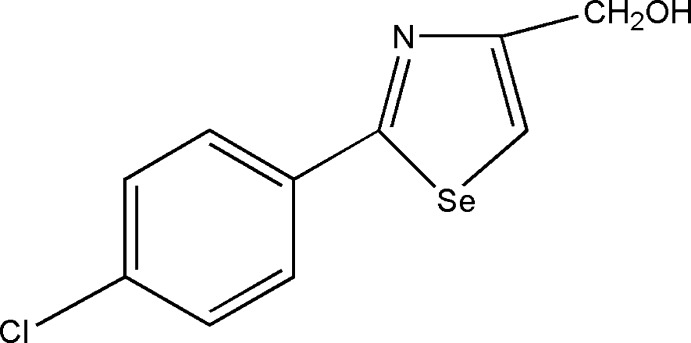


## Experimental

### 

#### Crystal data

C_10_H_8_ClNOSe
*M* *_r_* = 272.58
Monoclinic, 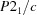

*a* = 14.8150 (15) Å
*b* = 4.5707 (4) Å
*c* = 14.9123 (14) Å
β = 96.642 (1)°
*V* = 1003.01 (16) Å^3^
*Z* = 4
Mo *K*α radiation
μ = 3.97 mm^−1^
*T* = 298 K
0.35 × 0.32 × 0.15 mm

#### Data collection

Bruker SMART APEX CCD diffractometer
Absorption correction: multi-scan (*SADABS*; Bruker, 2007 ▶) *T*_min_ = 0.337, *T*_max_ = 0.587
4466 measured reflections
1742 independent reflections
1318 reflections with *I* > 2σ(*I*)
*R*_int_ = 0.059

#### Refinement

*R*[*F* ^2^ > 2σ(*F* ^2^)] = 0.041
*wR*(*F* ^2^) = 0.104
*S* = 1.01
1742 reflections
128 parameters
H-atom parameters constrained
Δρ_max_ = 0.45 e Å^−3^
Δρ_min_ = −0.45 e Å^−3^

### 

Data collection: *SMART* (Bruker, 2007 ▶); cell refinement: *SAINT* (Bruker, 2007 ▶); data reduction: *SAINT*; program(s) used to solve structure: *SHELXS97* (Sheldrick, 2008 ▶); program(s) used to refine structure: *SHELXL97* (Sheldrick, 2008 ▶); molecular graphics: *SHELXTL* (Sheldrick, 2008 ▶); software used to prepare material for publication: *SHELXTL*.

## Supplementary Material

Crystal structure: contains datablock(s) I, global. DOI: 10.1107/S1600536812048088/qk2047sup1.cif

Structure factors: contains datablock(s) I. DOI: 10.1107/S1600536812048088/qk2047Isup2.hkl

Supplementary material file. DOI: 10.1107/S1600536812048088/qk2047Isup3.cml

Additional supplementary materials:  crystallographic information; 3D view; checkCIF report

## Figures and Tables

**Table 1:** Hydrogen-bond geometry (Å, °)

*D*—H⋯*A*	*D*—H	H⋯*A*	*D*⋯*A*	*D*—H⋯*A*
O1—H1⋯N1^i^	0.82	2.07	2.891 (5)	174

Symmetry code: (i) 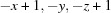.

**Fig. 1.**
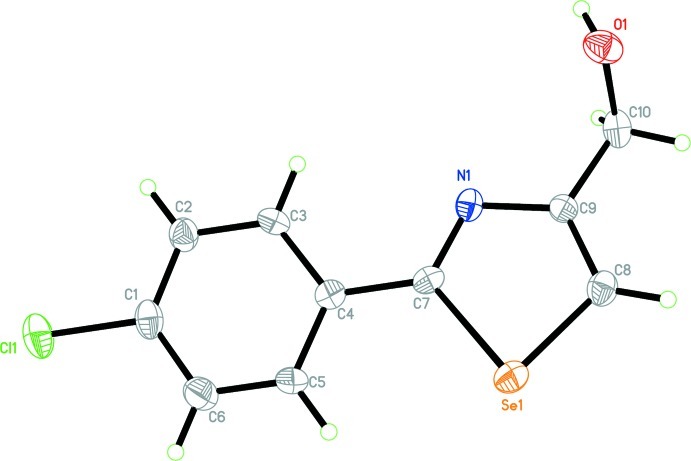
The molecular structure of the title compound with 50% probability displacement ellipsoids.

**Fig. 2.**
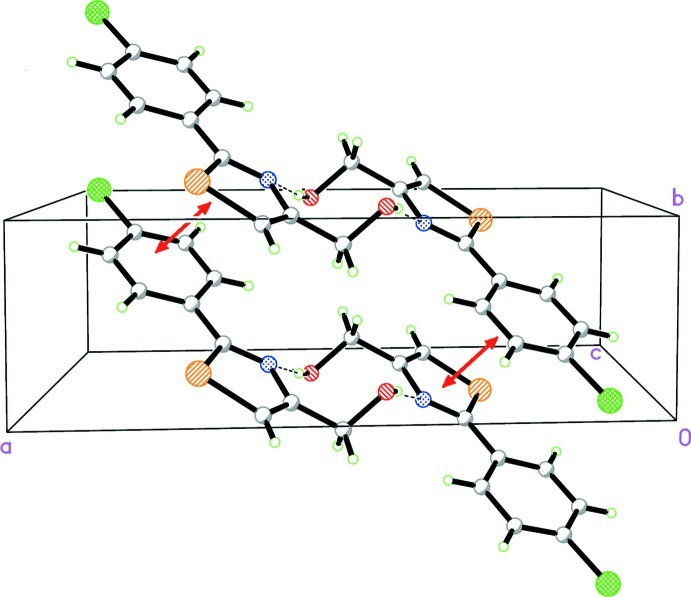
Packing diagram of the title compound indicating H-bonds by dashed lines and π-π-stacking by red double arrows (see Table 1 and text).

## supplementary crystallographic information

### Comment

It has well been confirmed that selenium-containing heterocyclic compounds are of considerable biochemical and pharmacological relevance. Thus, derivatives of selenazole have been extensively studied not only because of their interesting reactivities but also because of their pharmaceutical applications (Koketsu & Ishihara, 2003; Geisler *et al.*, 2004). Interested in this field, the title compound, a derivative of selenazole, was prepared and its crystal structure presented (Fig. 1). In the title compound, C_10_H_8_NOClSe, the dihedral angle between the nearly planar benzene and selenazole rings is 11.4 (3)°. The dihedral angle between the selenazole ring and the hydroxymethyl group, defined by C9—C10–O1, is 63.8 (3)°. All the bond lengths and angles are normal and correspond to those observed in the related compounds (Shen *et al.*, 2011; Shi & Zhao, 2007). In the crystal structure, pairs of molecules are disposed about an inversion center, generating dimers linked by intermolecular O—H···N hydrogen bonds (Fig. 2 and Table 1). The aromatic parts of the molecules are stacked along [010] (*b* = 4.5707 (4) Å) in a roof tile-like fashion enabling slipped π-π-stacking interactions. The face-to-face distance of the aromatic parts of the molecules (Se1, N1, C1 – C9) is 3.57 Å and their slippage 2.85 Å approximately parallel to the ring-to-ring bond C4—C7 (Fig. 2). This brings Cl1 in a position above/below the ring centroid of an adjacent benzene ring (Cl···centroid (C1 – C6) = 3.68 Å).

### Experimental

2-(4-Chlorophenyl)-4-chloromethyl-1,3-selenazole (0.01 mol) (Shafiee *et al.*, 1979) was added to dilute sulfuric acid (75 ml, 3.5 mol L^-1^) and heated at reflux for 8 h. The solution was made alkaline with dilute sodium hydroxide and extracted with chloroform. The organic layer was dried with Na_2_SO_4_, filtered, and evaporated and the obtained solid was recrystallized from ethanol. Clear block-shaped crystals were obtained.

### Refinement

C-bonded H atoms were placed in calculated positions and thereafter treated as riding, C—H = 0.93 and 0.97 Å, *U*_iso_(H) = 1.2*U*_eq_(C). The hydroxyl H atom was refined with AFIX 147 of program *SHELXL97* (Sheldrick, 2008), O—H = 0.82 Å, *U*_ĩso_(H) = 1.5*U*_eq_(O).

### Crystal data

**Table:**

C_10_H_8_ClNOSe	*F*(000) = 536
*M**_r_* = 272.58	*D*_x_ = 1.805 Mg m^−^^3^
Monoclinic, *P*2_1_/*c*	Mo *K*α radiation, λ = 0.71073 Å
Hall symbol: -P 2ybc	Cell parameters from 1928 reflections
*a* = 14.8150 (15) Å	θ = 2.8–27.1°
*b* = 4.5707 (4) Å	µ = 3.97 mm^−^^1^
*c* = 14.9123 (14) Å	*T* = 298 K
β = 96.642 (1)°	Block, white
*V* = 1003.01 (16) Å^3^	0.35 × 0.32 × 0.15 mm
*Z* = 4	

### Data collection

**Table:**

Bruker SMART APEX CCD diffractometer	1742 independent reflections
Radiation source: fine-focus sealed tube	1318 reflections with *I* > 2σ(*I*)
Graphite monochromator	*R*_int_ = 0.059
π and ω scans	θ_max_ = 25.0°, θ_min_ = 2.8°
Absorption correction: multi-scan (*SADABS*; Bruker, 2007)	*h* = −15→17
*T*_min_ = 0.337, *T*_max_ = 0.587	*k* = −5→5
4466 measured reflections	*l* = −17→14

### Refinement

**Table:**

Refinement on *F*^2^	Primary atom site location: structure-invariant direct methods
Least-squares matrix: full	Secondary atom site location: difference Fourier map
*R*[*F*^2^ > 2σ(*F*^2^)] = 0.041	Hydrogen site location: inferred from neighbouring sites
*wR*(*F*^2^) = 0.104	H-atom parameters constrained
*S* = 1.01	*w* = 1/[σ^2^(*F*_o_^2^) + (0.0525*P*)^2^] where *P* = (*F*_o_^2^ + 2*F*_c_^2^)/3
1742 reflections	(Δ/σ)_max_ < 0.001
128 parameters	Δρ_max_ = 0.45 e Å^−^^3^
0 restraints	Δρ_min_ = −0.45 e Å^−^^3^

### Fractional atomic coordinates and isotropic or equivalent isotropic displacement parameters (Å^2^)

**Table:**

	*x*	*y*	*z*	*U*_iso_*/*U*_eq_	
Se1	0.74166 (3)	0.13939 (10)	0.30636 (3)	0.0423 (2)	
Cl1	0.93843 (9)	1.0498 (3)	0.66855 (9)	0.0586 (4)	
O1	0.4348 (2)	−0.0090 (8)	0.3910 (2)	0.0465 (8)	
H1	0.4149	−0.0297	0.4396	0.070*	
N1	0.6330 (2)	0.1313 (7)	0.4388 (2)	0.0313 (8)	
C1	0.8698 (3)	0.8234 (9)	0.5942 (3)	0.0394 (11)	
C2	0.7838 (3)	0.7563 (10)	0.6124 (3)	0.0401 (11)	
H2	0.7612	0.8350	0.6628	0.048*	
C3	0.7307 (3)	0.5702 (9)	0.5551 (3)	0.0352 (10)	
H3	0.6722	0.5232	0.5672	0.042*	
C4	0.7644 (3)	0.4523 (9)	0.4790 (2)	0.0308 (10)	
C5	0.8507 (3)	0.5313 (11)	0.4624 (3)	0.0410 (11)	
H5	0.8735	0.4589	0.4112	0.049*	
C6	0.9042 (4)	0.7147 (11)	0.5196 (3)	0.0503 (13)	
H6	0.9626	0.7637	0.5078	0.060*	
C7	0.7083 (3)	0.2511 (9)	0.4200 (2)	0.0285 (9)	
C8	0.6372 (3)	−0.0813 (9)	0.2973 (3)	0.0373 (11)	
H8	0.6171	−0.1989	0.2481	0.045*	
C9	0.5933 (3)	−0.0506 (9)	0.3712 (2)	0.0293 (9)	
C10	0.5071 (3)	−0.2087 (10)	0.3850 (3)	0.0410 (11)	
H10A	0.4912	−0.3417	0.3349	0.049*	
H10B	0.5167	−0.3236	0.4399	0.049*	

### Atomic displacement parameters (Å^2^)

**Table:**

	*U*^11^	*U*^22^	*U*^33^	*U*^12^	*U*^13^	*U*^23^
Se1	0.0457 (4)	0.0490 (4)	0.0337 (3)	−0.0017 (2)	0.0118 (2)	−0.0033 (2)
Cl1	0.0543 (9)	0.0538 (9)	0.0629 (8)	−0.0135 (6)	−0.0131 (6)	−0.0081 (6)
O1	0.037 (2)	0.060 (2)	0.0427 (18)	0.0018 (18)	0.0058 (15)	0.0102 (16)
N1	0.034 (2)	0.029 (2)	0.0299 (17)	0.0012 (16)	−0.0013 (15)	−0.0014 (14)
C1	0.042 (3)	0.031 (3)	0.042 (2)	−0.002 (2)	−0.008 (2)	0.0014 (19)
C2	0.049 (3)	0.037 (3)	0.034 (2)	−0.008 (2)	0.005 (2)	−0.0008 (19)
C3	0.027 (3)	0.041 (3)	0.039 (2)	−0.004 (2)	0.0053 (19)	0.0034 (19)
C4	0.035 (3)	0.029 (2)	0.028 (2)	0.0021 (19)	0.0035 (18)	0.0047 (16)
C5	0.027 (3)	0.046 (3)	0.052 (3)	−0.002 (2)	0.013 (2)	−0.009 (2)
C6	0.036 (3)	0.047 (3)	0.069 (3)	−0.004 (2)	0.010 (3)	−0.005 (3)
C7	0.030 (3)	0.029 (2)	0.0265 (19)	0.0094 (19)	0.0022 (17)	0.0039 (17)
C8	0.040 (3)	0.039 (3)	0.032 (2)	0.004 (2)	−0.0018 (19)	−0.0006 (18)
C9	0.027 (2)	0.029 (2)	0.030 (2)	0.0053 (18)	−0.0034 (18)	0.0012 (17)
C10	0.042 (3)	0.039 (3)	0.039 (2)	−0.007 (2)	−0.004 (2)	−0.002 (2)

### Geometric parameters (Å, º)

**Table:**

Se1—C8	1.839 (5)	C3—H3	0.9300
Se1—C7	1.889 (4)	C4—C5	1.378 (6)
Cl1—C1	1.753 (4)	C4—C7	1.463 (6)
O1—C10	1.419 (6)	C5—C6	1.380 (6)
O1—H1	0.8200	C5—H5	0.9300
N1—C7	1.303 (5)	C6—H6	0.9300
N1—C9	1.383 (5)	C8—C9	1.350 (6)
C1—C2	1.367 (6)	C8—H8	0.9300
C1—C6	1.370 (6)	C9—C10	1.502 (6)
C2—C3	1.385 (6)	C10—H10A	0.9700
C2—H2	0.9300	C10—H10B	0.9700
C3—C4	1.399 (5)		
			
C8—Se1—C7	84.78 (19)	C1—C6—C5	118.8 (5)
C10—O1—H1	109.5	C1—C6—H6	120.6
C7—N1—C9	113.5 (4)	C5—C6—H6	120.6
C2—C1—C6	121.5 (4)	N1—C7—C4	125.2 (4)
C2—C1—Cl1	119.6 (4)	N1—C7—Se1	113.5 (3)
C6—C1—Cl1	118.9 (4)	C4—C7—Se1	121.3 (3)
C1—C2—C3	119.5 (4)	C9—C8—Se1	111.3 (3)
C1—C2—H2	120.3	C9—C8—H8	124.3
C3—C2—H2	120.3	Se1—C8—H8	124.3
C2—C3—C4	120.4 (4)	C8—C9—N1	116.9 (4)
C2—C3—H3	119.8	C8—C9—C10	123.9 (4)
C4—C3—H3	119.8	N1—C9—C10	119.1 (4)
C5—C4—C3	118.1 (4)	O1—C10—C9	111.1 (4)
C5—C4—C7	122.0 (4)	O1—C10—H10A	109.4
C3—C4—C7	119.9 (4)	C9—C10—H10A	109.4
C6—C5—C4	121.8 (4)	O1—C10—H10B	109.4
C6—C5—H5	119.1	C9—C10—H10B	109.4
C4—C5—H5	119.1	H10A—C10—H10B	108.0
			
C6—C1—C2—C3	1.1 (7)	C3—C4—C7—N1	−11.4 (6)
Cl1—C1—C2—C3	−178.0 (3)	C5—C4—C7—Se1	−11.1 (6)
C1—C2—C3—C4	−0.2 (7)	C3—C4—C7—Se1	169.0 (3)
C2—C3—C4—C5	−1.1 (6)	C8—Se1—C7—N1	0.0 (3)
C2—C3—C4—C7	178.7 (4)	C8—Se1—C7—C4	179.7 (3)
C3—C4—C5—C6	1.7 (7)	C7—Se1—C8—C9	0.3 (3)
C7—C4—C5—C6	−178.2 (5)	Se1—C8—C9—N1	−0.6 (5)
C2—C1—C6—C5	−0.6 (7)	Se1—C8—C9—C10	−178.4 (3)
Cl1—C1—C6—C5	178.5 (4)	C7—N1—C9—C8	0.6 (5)
C4—C5—C6—C1	−0.8 (7)	C7—N1—C9—C10	178.6 (4)
C9—N1—C7—C4	−180.0 (4)	C8—C9—C10—O1	−117.4 (4)
C9—N1—C7—Se1	−0.3 (4)	N1—C9—C10—O1	64.8 (5)
C5—C4—C7—N1	168.5 (4)		

### Hydrogen-bond geometry (Å, º)

**Table:**

*D*—H···*A*	*D*—H	H···*A*	*D*···*A*	*D*—H···*A*
O1—H1···N1^i^	0.82	2.07	2.891 (5)	174

Symmetry code: (i) −*x*+1, −*y*, −*z*+1.
